# Carcinoma of Pancreatobiliary Origin

**DOI:** 10.1155/2020/4323687

**Published:** 2020-08-05

**Authors:** Darina Kohoutová, Jan Bureš, Sameer Zar, Ondřej Urban

**Affiliations:** ^1^The Royal Marsden Hospital NHS Foundation Trust, London, UK; ^2^2nd Department of Internal Medicine-Gastroenterology, Charles University Faculty of Medicine in Hradec Kralove and University Hospital, Hradec Kralove, Czech Republic; ^3^2nd Department of Internal Medicine-Gastroenterology and Geriatrics, Faculty of Medicine and Dentistry, Palacký University Olomouc and University Hospital Olomouc, Czech Republic

Cancers of the pancreas and biliary tree remain one of the most dreadful oncological diagnoses with a very poor prognosis. Our special issue focused on the management of precancerous conditions and efforts to find new biomarkers which would enable precise and early diagnosis of these deadly cancers. We also focused on the role of diagnostic and therapeutic endoscopy, treatment of established carcinomas, and effective palliation.

Pancreatic cysts including mucinous ones and solid-cystic pseudopapillary tumours belong to precancerous lesions of a pancreatic adenocarcinoma. Despite all the progress in this field, including publishing the recent guidelines [1, 2], our clinical decision-making remains limited and further research, which will stratify the risk, is needed. Precursors to cholangiocarcinoma have been defined; nevertheless, these are rarely diagnosed, and precise characteristics and features of their progression into cancer are yet to be assessed [3, 4]. Management of indeterminate biliary stenoses is difficult in the clinical practice and evaluation of bile acids in the liver bile might help to differentiate between benign and malignant biliary stenoses [5].

Endoscopy plays an important role in the diagnosis of extrahepatic cholangiocarcinoma and pancreatic tumours. Endoscopic ultrasound (with fine-needle aspiration or fine-needle biopsy) and endoscopic retrograde cholangiopancreatography are essential methods for obtaining the tissue diagnosis. Cholangioscopy is being used for direct visualization of biliary intraductal processes and to aquire suspicious/malignant tissue when classical methods (including cytology and biopsies) have failed [6, 7].

Tailored surgical and oncological treatment should be available to every patient if this is clinically appropriate. Still, only 20% of patients with pancreatic ductal adenocarcinoma have resectable or borderline resectable pancreatic cancer at diagnosis. Neoadjuvant treatment is being evaluated in this group of patients [8]. Chemotherapy regimens have shown incremental survival gains in those where surgery is not an option, but more progress in this area is needed [9]. The critical situation in the cholangiocarcinoma group of patients remains similar.

Tailored palliation plays a crucial role in pancreatic cancer and cholangiocarcinoma, taking into account, that these cancers are usually diagnosed in late stages. Effective biliary drainage belongs to the cornerstones of palliative interventions, yet drainage of especially hilar cholangiocarcinoma can be challenging. Drainage of nonatrophic segments should be planned in an optimal scenario with a magnetic resonance cholangiography, and appropriate stents should be used (plastic or uncovered metal stents) [10].

The guest editors will appreciate if the readers benefit from reading this special issue which provides an overview of the most up-to-date knowledge from the world-leading experts.

*Darina Kohoutová* *Jan Bureš* *Sameer Zar* *Ondřej Urban*
